# Influence of the Aryl Hydrocarbon Receptor Activating Environmental Pollutants on Autism Spectrum Disorder

**DOI:** 10.3390/ijms22179258

**Published:** 2021-08-26

**Authors:** Hevna Dhulkifle, Abdelali Agouni, Asad Zeidan, Mohammed Saif Al-Kuwari, Aijaz Parray, Mohamed Tolefat, Hesham M. Korashy

**Affiliations:** 1 Department of Pharmaceutical Sciences, College of Pharmacy, QU Health, Qatar University, Doha 2713, Qatar; hevnakifly@gmail.com (H.D.); aagouni@qu.edu.qa (A.A.); 2 Biomedical and Pharmaceutical Research Unit (BPRU), QU Health, Qatar University, Doha 2713, Qatar; a.zeidan@qu.edu.qa; 3 Department of Biomedical Sciences, College of Medicine, QU Health, Qatar University, Doha 2713, Qatar; 4 Environmental and Municipal Studies Institute, Ministry of Municipality and Environment, Doha 2713, Qatar; msakuwari@mme.gov.qa; 5 The Neuroscience Institute, Hamad General Hospital, Doha 2713, Qatar; AParray@hamad.qa; 6 Department of Clinical Services, Shafallah Center for Children with Disabilities, Doha 2713, Qatar; mohamed.tolefat@shafallah.org.qa

**Keywords:** autism, environmental pollutants, AhR, CYP1A1, epigenetic modifications, polymorphism

## Abstract

Autism spectrum disorder (ASD) is an umbrella term that includes many different disorders that affect the development, communication, and behavior of an individual. Prevalence of ASD has risen exponentially in the past couple of decades. ASD has a complex etiology and traditionally recognized risk factors only account for a small percentage of incidence of the disorder. Recent studies have examined factors beyond the conventional risk factors (e.g., environmental pollution). There has been an increase in air pollution since the beginning of industrialization. Most environmental pollutants cause toxicities through activation of several cellular receptors, such as the aryl hydrocarbon receptor (AhR)/cytochrome P450 (CYPs) pathway. There is little research on the involvement of AhR in contributing to ASD. Although a few reviews have discussed and addressed the link between increased prevalence of ASD and exposure to environmental pollutants, the mechanism governing this effect, specifically the role of AhR in ASD development and the molecular mechanisms involved, have not been discussed or reviewed before. This article reviews the state of knowledge regarding the impact of the AhR/CYP pathway modulation upon exposure to environmental pollutants on ASD risk, incidence, and development. It also explores the molecular mechanisms involved, such as epigenesis and polymorphism. In addition, the review explores possible new AhR-mediated mechanisms of several drugs used for treatment of ASD, such as sulforaphane, resveratrol, haloperidol, and metformin.

## 1. Introduction

Autism spectrum disorder (ASD) is a disease that affects the development, communication, and behavior of an individual. It usually evolves during early childhood and persists throughout the lifetime of the affected individual, causing social and behavioral impairments. These defects may impede individuals with their day-to-day lives and make them dependent on the people around them [1]. Earlier understanding of autism was limited to a more severe variety of the spectrum, which resulted in pronounced speech defects, learning disabilities, and below-average IQ. Consequently, high-functioning individuals with variants of autism were misdiagnosed or underdiagnosed [1]. ASD is now used as an umbrella term encapsulating a large variation in developmental and behavioral disorders, including autism disorder, Asperger’s disorder, childhood disintegrative disorder, and pervasive developmental disorder—not otherwise specified [2].

Prevalence of autism has risen in recent years, from 0.4% in the 1960s–1970s to approximately 1.9%, affecting approximately 1 in 54 children in 2016 [3]. Although part of the increase could be attributed to better awareness and early detection, diagnostic drift does not completely account for the astounding numbers [4]. Individuals with ASD have symptoms and signs that are characteristically different from each other. Nonetheless, the diagnosis of ASD is dependent on two core domains, the social communication deficit and repetitive–restrictive behavior [5]. According to the Diagnostic and Statistical Manual of Mental Disorders criteria for ASD (DSM-5), social communication deficits are characterized by the impairment of socioemotional reciprocity, non-verbal communicative behaviors, and poor ability to develop, maintain, and understand relationships [6]. Manifestations of the repetitive–restrictive behavior include stereotyped or repetitive motor movements, inflexible adherence to routines or ritualized patterns of verbal and non-verbal behavior, abnormally high restricted or fixated interests, and hyper-reactivity or hypo-reactivity to sensory input [7].

The etiology of ASD is complex with several neurobiological factors contributing to its development, including genetic, epigenetic, and environmental factors [8,9]. Genetic influences have been recognized as the most likely cause of autism, in which approximately 50% ASD cases are inherited [10]. De novo mutations in the parent gametes have been established as a cause of sporadic occurrence of autism, accounting for nearly 30% of all autism cases in males and 45% in females [11]. Epigenetic causes, such as DNA methylation, genomic imprinting, and histone modification also instigate ASD [12,13,14]. Prenatal factors that induce changes in inflammatory cytokines, such as diabetes, Rubella, cytomegalovirus infections, and persistent fever during pregnancy are risk factors leading to autism [15]. In this regard, it has been shown that gene expression and plasma levels of several proinflammatory cytokines, prostaglandins, chemokines, and inflammation/oxidative stress-related proteins are highly elevated and strongly correlated with clinical features of children with ASD when compared with age-matched control [16,17,18,19]. Alternatively, a link between low thyroid hormone levels during pregnancy and an increased risk for autistic traits has been reported [20]. 

Although some factors contributing to the development of autism have been identified and characterized, these traditionally recognized risk factors alone cannot explain the rapid increase of autism incidence worldwide. There is extensive, ongoing research taking place to examine the possibility of the involvement of other risk factors, particularly exposure to environmental pollutants. The U.S. Environmental Protection Agency has reported an increase in the role of environmental factors in ASD incidence and development more than what was previously thought. In the past decades, uncontrolled industrialization and development of several human activities resulted in the emission of pollutants, such as heavy metals, aromatic hydrocarbons, dioxins, and phthalates, into the air, water, and soil, finally resulting in a massive environmental pollution. Consequently, people have been exposed to a mixture of these environmental chemicals/pollutants unknowingly, continually, and chronically. Co-contamination with complex mixtures of environmental pollutants is a common environmental problem with multiple biological consequences, particularly to the enzyme systems and metabolism in the body. The American and Canadian agencies for Environmental Protection Act rank heavy metals, polycyclic aromatic hydrocarbons (PAHs), and other environmental pollutants among the most hazardous and toxic substances in the environment [21,22]. The increase in everyday exposure and accumulation of environmental toxins through air, water, soil, and food have been revealed to play a critical role in the pathogenesis of several diseases, such as cardiovascular diseases [23,24], cancer [25,26], respiratory diseases [26,27], diabetes mellitus [28], and neurological diseases [29].

## 2. Environmental Pollution and ASD

Brain development during fetal and infant period is a critical stage and, consequently, toxicants may affect and alter brain development most dramatically at this stage compared to adults [30]. Prenatal exposure to air pollution has been associated with a range of adverse birth consequences, particularly with brain structural and neuropsychological abnormalities in childhood. These abnormalities include brain immune function, synaptic plasticity, brain circuitry, and stem cell development, in addition to selective exclusion of excess neuronal synapses [31,32,33]. The hypothesis that ASD development in early childhood or during adulthood is a result of prenatal exposure to environmental pollutants has been tested in human and animal studies. In that, a growing body of literature has now established an increase in the risk, incidence, and severity of ASD development with exposure to environmental pollutants during pregnancy and early childhood. 

Carter and Blizard have conducted a study to examine ASD gene-environment interaction and showed that approximately 67,861 chemical–gene interactions affected the autism susceptibility genes (ASGs), among which 4428 environmental toxins and chemicals affected one or more ASG, suggesting that ASGs are targets for environmental toxins [34]. For example, autism has been linked with organochlorine insecticides via disruption of pre- and post-synaptic dopamine, GABA, and glutamate function [34]. Another example of a close relationship between genes and the environment is the toxicogenomic effect of copper metal on reducing ProSAP/Shank protein levels in the brain, and decreasing the expression of the N-methyl-D-Aspartate receptor (GRIN1) in the excitatory synapse associated with autism [35]. A recent nationwide case-control study conducted in Denmark, which included 15,387 children with autism and 68,139 healthy age- and sex-matched children, revealed that exposure to air pollution in early stages of infancy, but not during pregnancy, contributed to increased risk of ASD [33]. 

Particulate matters (PMs) are small air pollutants composed of microscopic solid particles or liquid droplets that can easily enter the lungs and cause diseases, including autism [36]. PMs are one of the most investigated air pollutants with regards to autism as they carry all hazardous particles suspended in the air, including organic compounds, diesel exhaust, polycyclic aromatic hydrocarbons, endotoxins, and reactive heavy metals [37]. Traffic, domestic heating, and industrial emission are major contributors to ambient PM concentrations [38]. For example, traffic diesel exhaust has been estimated to contribute to more than 35% of air PM [39] and, thus, increased autism incidence was positively correlated with proximity to highways and exposure to PM_10_ during the third trimester, with an estimated odds ratio of 1.36 [32,40,41,42]. Although several studies have shown an association between PM and ASD; specific evidence for individual constituents is conflicting. A case-control study in Shanghai reported an increased risk of developing ASD among children below 3 years who were exposed to PMs [43]. Systematic reviews and meta-analysis studies conducted to review epidemiological literature on the impact of PM exposure on ASD showed a strong evidence of association for prenatal and postnatal exposure to PM_2.5_, with little evidence for PM_10_ [44,45,46]. Geng and coworkers have demonstrated a significant correlation between autism severity and the PM_2.5_ serum levels of children with autism [47]. At the experimental level, exposure of neonatal male Sprague–Dawley rats to PM_2.5_ caused autism-like behavioral abnormalities, such as communication deficits, decreased social interaction, and aversion to unfamiliar objects [48]. This delirious effect could be possibly attributed to induction of neuroinflammation, dysregulation of immune system, mutation of a pivotal gene involved in formation, maturation, and maintenance of synapses [48], and DNA methylation and oxidative stress [49]. 

Heavy metals are stable air pollutants that are neither created nor biodegradable, making the exposure to metallic elements a matter of growing concern [50]. Heavy metals are known as neurodevelopmental toxicants causing fetal damage and neurological diseases, including autism. Several case-control studies revealed that chronic exposure to inorganic mercury resulted in a staggering 60% increase in susceptibility to autism. Recent systematic reviews and meta-analysis studies showed that ASD patients exhibited higher concentrations of heavy metals, such as antimony, mercury, lead, in their hair and blood, which were positively associated with an increased risk of autism [46,51]. In addition, the higher levels of heavy metals in children with autism than matched controls were positively correlated with maternal fish consumption, maternal use of dental amalgam, residing near gasoline stations, and usage of aluminum pans [52]. These results imply that development index of countries significantly influences the overall concentrations of heavy metal toxicity between patients with ASD and control subjects, which support the theory that environmental pollution is a contributory factor to ASD. 

Organic pollutants are long-lived toxic substances in the environment. They are generally divided into persistent and non-persistent, where non-persistent organic pollutants (NPOPs) are toxins that do not remain in the human body, but still harmfully affect various physiological pathways. The link between exposure to NPOPs, such as phthalate and bisphenol A (BPA), and autism development in children, has been reported in several studies that showed higher levels of phthalates and BPA compounds in the blood and urine of children with autism compared to healthy subjects [53,54,55]. On the contrary, other studies demonstrated that prenatal exposure to phthalates during the second and third trimesters of pregnancy was not associated with an increased risk of autism in children from this cohort [56]. On the other hand, persistent organic pollutants (POPs), such as organochlorine pesticides, polychlorinated biphenyls (PCBs), perfluoroalkyl substances (PFAS), polychlorinated dibenzofurans (PCDFs), and polycyclic aromatic hydrocarbons (PAHs) are generated from anthropogenic activities that resulted in the accumulation of these toxic substances in the soil, air, and water [34,57]. The direct link between exposure to POPs and autism has been reported. A population-based case-control study aimed to determine the impact of prenatal exposure to PCBs during pregnancy on autism demonstrated that elevated levels of PCB138/158, 155, and 170, were associated with higher risk of development of ASD, probably through specific gene modulations [58]. In addition, exposure to PCBs causes 15q11-q13 duplication autism spectrum disorder and development of autistic traits [59,60,61,62]. Further supporting evidence for the association of environmental pollutants and autism is the observation that elevated levels of 2,3,7,8,-tetrachlorodibenzo-p-dioxin (TCDD), a well-known PAH, in breast milk, increased autistic traits of 3-year-old children in Vietnam [63]. Importantly, these POPs and PAHs are known to exhibit their toxic effects on the human body through the activation of a cytosolic protein known as the aryl hydrocarbon receptor (AhR) [21], suggesting high possibility that the AhR pathway could mediate increased autism development and incidence. The next part of the review discusses recent advances and studies, highlighting the impact and role of the AhR pathway in the incidence of autism.

## 3. Aryl Hydrocarbon Receptor Pathway and ASD

### 3.1. Aryl Hydrocarbon Receptor Pathway

**Figure 1:**
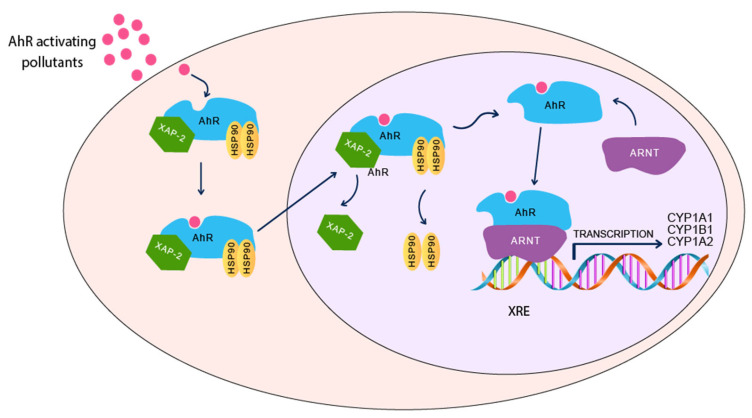
AhR/CYP1 activation signaling pathway.

AhR is a ligand-activated transcription factor that belongs to basic-helix-loop-helix (bHLH)/Per-ARNT-Sim (PAS) family, which is involved in the regulation of cell differentiation, proliferation, and cancer imitation [64,65]. AhR plays an important role in various physiological pathways, including host defense, immunity, stem cell maintenance, cell differentiation, and xenobiotic metabolism [66]. It was initially believed that AhR is activated only by a group of environmental pollutants, such as polycyclic aromatic hydrocarbons (PAHs). However, it is now reported that several non-PAHs, such as ketoconazole [67] and heavy metals [22,68], could modulate AhR. Human AhR is found in the cytosol in complex with XAP2, an inhibiting chaperone protein Heat Shock Protein 90 (HSP90) and is protected from degradation by its association with p23 [69]. Activation of AhR upon binding to its ligand results in its translocation from the cytosol into to the nucleus and dissociation from HSP90 (Figure 1). The activated AhR heterodimerizes with a transcription factor, known as the AhR nuclear translocator (ARNT), inside the nucleus. The resulting complex binds to specific DNA sequence, xenobiotic response element (XRE), located on the enhancer regions of specific genes, leading to initiation of their transcriptional and translational expression [70]. Examples of these AhR regulated genes include, CYP1A1, CYP1A2, CYP1B1, and AhR repressor (AhRR). Induction of CYP1 genes is capable of bioactivating environmental toxicants and transforming them into their reactive moieties, such as epoxide, which can attack general macromolecules, such as RNA, DNA, and proteins of specific organs and tissues, by forming DNA adducts, inducing oxidative stress, forming genotoxic compounds and eventually resulting in tissue damage [71]. Activation of AhR is now known to be involved in the pathogenesis of several diseases, such as cancer [72,73], cardiovascular diseases [74], inflammatory diseases [75], atherosclerosis [76], and neurodegenerative disease [77].

### 3.2. Evidence of Involvement of AhR/CYP1A Pathway in Autism Development

Dioxin-like chemicals are well-known neurotoxic pollutants, where exposure to these chemicals has been linked with increased the risk of autism. Since these environmental toxicants target AhR to mediate their toxicities, it is highly possible that AhR could play a role in autism development during childhood; however, the links between AhR and autism are still not fully revealed. What supports this possibility is that AhR and its regulated genes, CYP1A1, CYP1A2, and CYP1B1, are highly and constitutively expressed in the placenta, which may be activated by exposure to environmental toxicants during pregnancy and, hence, increase the incidence of autism [78]. In addition, activation of the AhR/CYP1 results in DNA adduct formation and DNA strand breakage [79,80], considered as risk factors for the development of autism [80]. These results collectively indicate that AhR/CYP1 could play a role in ASD incidence. This section summarizes the most recent human and experimental studies (Table 1) and evidence for the potential role of AhR and its regulated genes, CYP1A1, CYP1B1, and CYP1A2 on autism development. 

#### 3.2.1. Human and Epidemiological Studies

Neuroinflammation has been hypothesized to contribute to autism development; for example, it was reported that the levels of pro-inflammatory cytokines are high in the blood and cerebrospinal fluid of patients with autism. A recent study on children with autism and age-matched healthy children showed elevated levels of AhR- mediated gene expressions of several inflammatory cytokines, such as interleukin-6 and signal transducer and activator of transcription 3 (STAT3) in children with autism, more than in healthy individuals [16]. This is supported by reports showing that STAT3 binds to its motif in the AhR promoter region; thus, activating AhR. There is a strong correlation between autism severity and the levels of vitamin D, in which children with autism are usually associated with vitamin D deficiency. Knowing that vitamin D is metabolized by CYP1B1, it is highly suggested that variation in CYP1B1 expression could play a role. The link between CYP1B1-mediated vitamin D deficiency and autism has been examined by El-Ansary and coworkers, who were the first to show that the plasma levels of CYP1B1 and vitamin D in 28 children with autism were 70% lower than their age- and sex-matched neurotypical children [81]. Although there is no other supporting study, it was postulated that decreased CYP1B1 levels in patients with ASD could be attributed to epigenetic silencing of the CYP1B1. 

Meta-analysis studies across two prospective pregnancy cohorts showed that the CYP1A1 gene expression was downregulated in umbilical cord blood from subjects with autism, suggesting its involvement in ASD etiology [82]. In addition, it has been reported in two separate studies that exposure of Vietnamese and Taiwanese pregnant women to dioxin, an AhR activator, was associated with increased neurodevelopmental deficits and autistic traits in children with ASD [63,83]. A cohort study examined the impact of prenatal exposure to PCDFs on autistic traits in middle- to late childhood using the Social Responsiveness Scale (SRS), and found that higher levels of PCDFs in maternal blood during pregnancy were associated with lower SRS scores in children, which resulted in greater autistic-like social traits [61]. A recent case-control study showed that elevated levels of POPs (PCBs, dioxins, PFAS), elements, and heavy metals in the amniotic fluids of children with autistic traits were associated with increased transactivation of AhR [61]. This study provides evidence that environmental pollutants can cross the placenta and, hence, increase the risk of toxicities and ASD. Although the levels of PFAS were lower in ASD cases compared to the control, this could be explained by the possible removal of some PFAS congeners during the process of extraction, as PFASs are high albumin-binding compounds [78]. 

#### 3.2.2. Experimental Animal Studies

A handful of animal studies has linked environmental pollutants to autism-like behavior through the AhR pathway. An in utero electroporation study by Kimura et al., conducted on pregnant C57BL/6N mice on gestational day 14, to transfect the neurons with constitutively active AhR vector plasmids, showed a constitutively activated AhR signaling [84]. This activation detrimentally affects neuronal migration during hippocampal development [84]. The cholinergic system is one of the pathways that has been investigated in neurotoxicity, ASD, and associated core symptoms [63,85,86]. Acetylcholinesterase (AChE) in the brain hydrolyzes the neurotransmitter acetylcholine (ACh) into acetyl-CoA and choline, which is a critical player in learning cognition and memory, especially during fetal and infant development stages. Dysregulation of AChE may have lasting detrimental effects on neural development contributing to autistic-like behavior [87]. Many dioxin-like compounds, such as TCDD, downregulate the transcription of AChE and suppress neuronal activity [88]. AhR activation via exposure to TCDD during the perinatal period of a rat model caused permanent brain damage and impaired the development of cerebellum of their offspring [89]. These toxic effects are believed to be attributed to a decrease in the levels of thyroid hormone, AChE, and monoamines levels, with an increase in gamma aminobutyric acid (GABA) levels in cerebellum of offspring [89]. Xie et al. have provided more evidence supporting the involvement of AChE, in that treatment of cultured SK-N-SH human-derived neurons with TCDD resulted in a significant decrease in enzymatic activity of AChE, whereas treatment with AhR inhibitor, CH223191 resulted in the restoration of the TCDD-mediated suppression of AChE [90], indicating that AChE is regulated, at least in part, through an AhR-dependent mechanism. 

Autism-like behavior, including anxiety, locomotor activity, repetitive behavior, and altered swimming pattern was induced in zebrafish when gestationally exposed to heavy metal lead, which was associated with upregulation of CYP1A [91]. On the other hand, Glazer et al. have studied the impact of developmental exposure to low levels of PCB126 on early- and later-life behavioral phenotypes in the zebrafish model system [92]. In this study, adult behavioral assays, including shoaling and the novel tank assay, showed that exposure to PCB126 had impaired short- and long-term habituation to the unfamiliar environment, and exhibited high anxiety-related behavior with no change in the larval locomotor activity. These autistic effects of PCB126 were associated with a significant induction of CYP1A in early stages of development, with no significant upregulation at adulthood [92], implying that activation of AhR in the early developmental stages due to exposure to POPs is linked with autistic traits. In addition, Colter et al. and coworkers have used high affinity *Ahr^b^Cyp1a2^(−/−)^* and poor affinity *Ahr^d^Cyp1a2^(−/−)^* knockout mice models to study the effect of developmental PCB exposure on autism development [93]. In their studies, they found that both high- and poor-affinity knockout mice displayed motor dysfunction when exposed to high PCB levels during gestation and lactation, with higher susceptibility to nigrostriatal dysfunction and motor deficit in high-affinity *Ahr^b^Cyp1a2^(−/−)^* knockout mice [93]. A follow-up study on the same mouse models has also established that high-affinity *Ahr^b^Cyp1a2^(−/−)^* mice exposed to PCBs displayed the highest levels of toxicity and variation in gene expression in the cerebellum and cortex, the two centers of the brain responsible for motor activity and memory [94]. These studies clearly provide strong evidence for the involvement of the CYP1A2 gene in the neurotoxicity caused by developmental exposure to PCBs.

**Table 1:** The role and mechanisms of AhR/CYP1 pathway in ASD development.

Gene	Study Model	Study Design	AhR/CYP1 Modulator	Effect on AhR/CYP1 Pathway	Effect on Autism Incidence & Development	References
***AhR***	Human	SK-N-SH human-derived neurons	TCDD	AhR activation → ↓AChE	↓ Neuronal activity	[90]
			CH223191	AhR inhibitor, CH223191 → ↑ AChE	↑ Neuronal activity	
		Pregnant amniotic fluids	PCBs & heavy metals	↑ AhR transactivation	↓ The levels of PFAS were lower in ASD cases compared to control.	[78]
	Rats	Perinatal exposure to TCDD	TCDD	Activation of AhR → ↓ AChE, monoamines, ↑ stimulation of GABA ↓ thyroid hormones, increase in TSH, decrease growth hormones in cerebellum of offspring	↑ Permanent brain damage. Impaired the development of cerebellum of their offspring	[89]
***CYP1A***	Human	Autistic subject		↓ CYP1A1 gene expression in umbilical cord blood	↑ ASD incidence compared to control	[82]
		Pregnant	Dioxin	↑ dioxin levels in maternal blood	↑ Neurodevelopmental deficits and autistic traits in the children with ASD	[63,83]
			PCDFs	↑ PCDFs levels in maternal blood	↑ Autistic traits in middle to late childhood using SRS	[61]
	Zebrafish	Developmental exposure to PCB126 on early- and later-life behavioral	PCB126	↑ CYP1A1 in early stages of development, with no significant upregulation at adulthood	Impaired short-term and long-term habituation to unfamiliar environment ↑ Anxiety-related behavior with no change in the larval locomotor activity	[91,92]
	Mice	Pregnant C57BL/6N	AhR plasmid transfection	Constitutive AhR activation	Affects neuronal migration during hippocampal development.	[84]
		High affinity *Cyp1a2^(−/−)^*	PCBs	CYP1A2 Knockout	↑ Motor dysfunction compared to wild-type mouse. ↑ Susceptibility to nigrostriatal dysfunction and motor deficit, and toxicity of the cerebellum and cortex.	[93,94]
***CYP1B1***	Human	Autistic children	Vitamin D deficiency	↓ CYP1B1 plasma levels by 70% through epigenetic silencing of CYP1B1	↓ Vitamin D by 60% → positively correlates with ASD	[81]

## 4. Molecular Mechanisms of AhR/CYP1A Regulation in ASD Development

**Figure 2:**
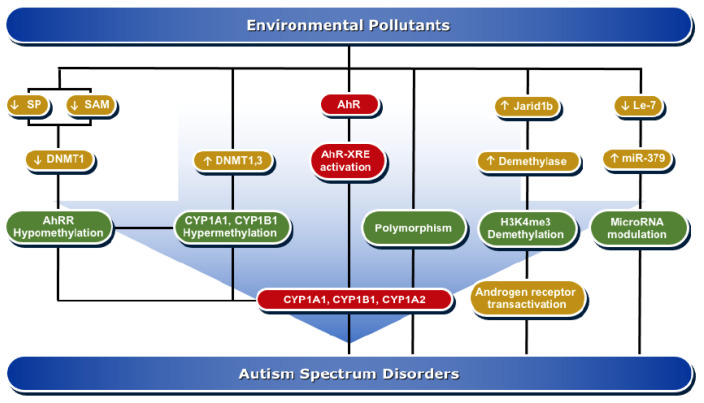
Graphical summary of AhR activation mechanisms in ASD development.

The interplay between genetics and environmental factors, mediated by AhR, and the cause for autism raises the question of how exactly changes in the AhR pathway is capable of contributing to ASD. Human neurodevelopmental diseases are well-linked to genetic disruptions. Although, the genetic and epigenetic mechanisms controlling ASD were recently reviewed by Yoon et al. [95] and others [96,97], these reviews did not discuss the impacts of genetic, epigenetic, and polymorphic regulations of AhR and CYP1 genes on ASD risk. This section discusses the most recent studies and evidence (Table 2) for the role and impact of epigenesis and polymorphism of AhR and regulated genes in autism development and risk. The roles of epigenesis and polymorphism are summarized in Figure 2. 

### 4.1. Epigenesis

Human neurodevelopmental diseases are well-linked to epigenetic disruptions. Epigenesis is an interesting area that gives insight into the machinery processes involved in disease development, including ASD. These machinery processes include DNA modification, histone tails modification, chromatin organization, and remodeling. Although some recent reviews have addressed the impact of transcriptional regulatory influences of environmental pollution and factors on the epigenetic mechanism of ASD etiology, the role of AhR and regulated genes was not discussed. This section discusses evidence for epigenetic changes in ASD through regulation of AhR/CYP1 pathway. 

#### 4.1.1. DNA Methylation

DNA methylation is one of the important mechanisms in epigenetics, in which cytosine is transformed into 5-methylcytosine by the transfer of a methyl group mediated by DNA methyltransferase (DNMTs) enzymes [98]. DNA methylation is pivotal in the regulation of gene expression, either by altering the recruitment of proteins or by hindering the binding of transcription factors to DNA. Both de novo hypermethylation and hypomethylation of the enhancer or promoter region of DNA during development are capable of changing the pattern of DNA methylation in the genome. This results in cell differentiation and development of a unique and stable DNA methylation pattern that can regulate tissue-specific gene transcription [99].

DNA methylation can affect brain tissue differentiation, nervous system development, and cause intellectual disorders, including autism. Mitchell et al. have reported that persistent exposure to organic pollutants, such as PCBs, causes epigenetic DNA methylation that is implicated in 15q11-q13 duplication autism spectrum disorder [59]. A genomic DNA study in postmortem individuals with ASD and in the cerebellum of BTBR T+tf/J autistic mice showed a significant increase in the expression levels of DNMT3a and DNMT3b as compared to non-autistic controls, which was positively correlated with the degree of DNA damage [100]. DNA methylation is one of the suggested mechanisms by which environmental pollutants, such as PCBs, are capable of inducing ASD development [101]. In that, the induction of oxidative DNA damage by AhR activation is aberrant DNA methylation [102]. An epigenome-wide study conducted in individuals perinatally exposed to PCBs and PCDFs compared to non-exposed subjects examined the methylation changes lasting to adulthood. The study showed differential DNA methylation for 20 CpGs mapped to 11 genes, including AhRR, CYP1B1, and CYP1A1 [102]. Men perinatally exposed to PCBs and PCDFs showed hypermethylation of CpG cg06264984 at CYP1B1, cg05549655 at CYP1A1, and cg17924476 in AHRR, with positive correlation with gestational levels of PCBs or PCDF toxic equivalency and exhibited hypomethylation of cg05575921 and cg21161138 in AhRR that were inversely related to PCB levels [102]. 

Several cohort studies had linked hypomethylation of AhRR and hypermethylation of CYP1A1 genes in cord blood of newborns with maternal smoking during pregnancy, indicating that epigenetic mechanisms are involved in the pathogenesis of diseases associated with in utero smoking exposure. For example, studies investigating maternal smoking during pregnancy had reported AhRR hypomethylation in the offspring’s cord blood mononuclear cells (CBMCs), buccal epithelium, placenta tissue [103,104], and was confirmed in the peripheral blood of their children at 17 years of age [105]. Additionally, a cohort study conducted in the Japanese population identified hypomethylated CpG sites cg05575921 and cg21161138 in AhRR and hypermethylated site cg05549655 in CYP1A1, in cord blood or newborn blood, due to prenatal tobacco smoke exposure [106]. However, whether these alterations in DNA methylation patterns in AhRR and CYP1A1 persist after mothers stopped smoking early during pregnancy remain controversial. While Miyake et al. showed that these DNA methylation patterns persist after smoking cessation [106], others have reported no significant differences between children from women who never smoked and those who stopped smoking after pregnancy [104]. The AhRR hypomethylation results in reduction in the ability of AhRR to compete with AhR to dimerize with the ARNT and, thereby, affect binding to XREs, leading to activation of AhR and the target gene, CYP1A1. These studies do not only indicate that the epigenetic postnatal stability of the DNA methylation of AhRR at 18 months is a mediator for long-term impacts in humans due to prenatal exposure to toxins [104], but also suggest DNA hypomethylation in the early development period can persist for a long period, and indicate possibility of AhR/CYP1A1 causing autism in individuals facilitated by the hypomethylation of its repressor gene by environmental toxins. 

In experimental animal models, researchers have studied the effects of pre-and postnatal exposure to a mixture of AhR activators, such as PCBs, PCDD, methylmercury (MeHg), and organochlorine pesticides, on hepatic, uterus, and brain DNA methylation in prepubertal female Sprague–Dawley rats. In these studies, researchers found that the AhR activators induced CYP1A1 activity, which was associated with a significant decrease in the global genome DNA methylation and the mRNA levels for DNMT1, DNMT3a, and DNMT3b in brain homogenate and brain areas, such as hypothalamus, hippocampus, and cortex [107,108]. Mechanistically, inhibition of DNA methylation by the AhR activators is mediated through downregulation of Sp1, a regulator of DNMT1 expression in the brain [109] and the reduction of S-adenosyl methionine (SAM) concentrations, universal methyl donor involved in DNA methylation [107].

A harmonious communication between various hormones is imperative to proper neurodevelopment. Since ASD is more prevalent in males than females, endocrine disruption is hypothesized to be a contributory factor to ASD. It has been found that DNA methylation is a very important player in sex-specific gene expression. PCBs have influenced sexual differentiation [110]. In one study, it was found that exposure of Sprague–Dawley rats to a mixture of PCBs, Aroclor 1221 on gestational days 1, 3, 16, and 18, caused a delay in onset of puberty in males and estrous cyclicity in females [111]. These effects were associated with significant increases in the gene expression of DNMT1 and ARNT in a sex-specific manner. Increased DNA methylation in response to PCB exposure alters endocrine function and gene expression in the brain, which influence the sexual development and sex-specific transcriptional profile in the brain [101,111]. These studies support the possibility that the AhR/CYP1 pathway is involved in ASD development through DNA methylation changes, which could persist in the offspring for 20 years or even more, leading to diseases, including autism. 

#### 4.1.2. Histone Modifications

Histones are building blocks of eukaryotic chromatin, which play a pivotal role in gene regulation. DNA wraps around these protein octamers, made of two each of H2A, H2B, H3, and H4 [112,113]. Tails protrude from the nucleosome H3 and H4, allowing post-translational modification to alter the histone interactions with DNA and other proteins. Histone acetylation or deacetylation, through histone acetyltransferases (HATs) and histone deacetylases (HDACs), and methylation, through methyltransferases, control genes of the developmental stage and, thus, the regulation of many physiological and disease-related pathways [114].

Epigenetic modifications of the histone either through acetylation or deacetylation mechanisms impact neurodevelopmental diseases, including autism. Blocking of histone deacetylation in the hippocampus leads to suppression of cognitive impairment and neurogenesis. This hypothesis is supported by the observation that valproic acid, a well-known inducer of autism, inhibits HDAC, causing hyperacetylation of the histone [115]. In an in vivo mouse model of autism, Shpyleva et al. demonstrated that the expressions of histone acetylation (H3K9ac and H3K56ac) and histone lysine 4 trimethylation (H3K4me3, H3K9me3, H3K27me3, and H4K20me3) in the cerebellum of BTBR T+tf/J autistic mice were not different from that of control C57BL/6J mice [100]. 

The higher incidence of ASD in males more than females suggests the role of prenatal exposure to male hormone androgens during brain development in animals and in humans [116]. In that, it has been reported that fetal testosterone levels were positively correlated with autistic traits, restricted interests and systemizing behaviors, and that reduction of the levels of androgens in individuals with ASD or animals would cause a significant decrease in their clinical symptoms [117,118]. In this context, several experimental [119] and epidemiological [120] studies have reported that exposure to AhR activators, such as PCBs, transactivates androgen receptor by enhancing the epigenetic demethylation of lysine 4 on histone H3 (H3K4me3) mediated by Jarid1b enzyme [121], causing mutations and, hence, ASD [122]. In addition, it was demonstrated that PCBs directly activate the XRE located on the androgen receptor promotor, along with androgen responsive element facilitating transactivation of androgen receptor target genes through the recruitment of Jarid1b [121,123], in which mutation of Jarid1b genes encoding for H3K4me3 demethylase results in autism [122]. These studies clearly support the hypothesis that early-life exposure to PCBs induces hyperandrogenization in the brain effects through AhR-mediated epigenetic mechanisms. 

#### 4.1.3. MicroRNAs

MicroRNAs (miRNAs) are a group of small noncoding RNAs that are approximately 22 nucleotides long. They are involved in the post-transcriptional regulation of gene expression by degrading their target mRNAs and, thus, modulating their translation [124]. They are capable of silencing mRNA by either cleavage of the mRNA strand, destabilization of the mRNA by shortening its poly(A) tail, or reducing efficiency of translation of the mRNA into proteins by ribosomes [125]. miRNA dysregulations are known to mediate pathogenesis of several human diseases, including ASD, and, thus, are considered a potential therapeutic target. 

Altered expression of miRNAs and their role in autism have been reviewed by Schepici et al., and others [126,127]; however, the effects of AhR/CYP on these miRNAs involved in autism have not been discussed. Toxicities of environmental pollutants, including PCBs and dioxins on autism, have been well characterized to be regulated by the AhR/CYP1 pathway, leading to the induction of a wide range of genes that express XREs on their promoters. However, the involvement of miRNAs in this regulation is unclear, particularly the effect of prenatal exposure to TCDD. In this context, it has been demonstrated that prenatal exposure of a mouse to TCDD caused the alteration of more than 100 miRNAs in fetal thymocytes [128]. Among these miRNAs, miR-379, which regulates brain neuronal development, was upregulated, whereas let-7, which regulates neuronal stem cell proliferation, was downregulated. Induction of miR-379 induces hypo-social behavior observed in autism patients. However, the regulation of miRNAs by the AhR/CYP1 pathway was not investigated in autism and warrants further investigation. 

### 4.2. Genetic Polymorphism

Gene polymorphism refers to the phenomenon where more than one allele occupies a gene’s locus within a population. Polymorphism refers to mutation of a gene in a single nucleotide (SNPs), or more. However, unlike any other mutation, an allele must occur in at least 1% of the population for that allele to be considered a polymorphism of its gene [129]. Polymorphism in genes results in a change in gene expression or the production of an altered form of a protein. These alterations can result in a cascade of changes that affect an individual’s physiology. 

The variation in the promoter region of several genes has been associated with ASD. Evidence of multiplicative interaction between a common environmental air pollutant, NO_2_, local traffic-related air pollution, and one of the functional promoter variants (rs1858830) in the MET receptor tyrosine kinase in patients with ASD was reported [32]. Fujisawa et al. examined the relationship between AhR-related gene polymorphisms and autism susceptibility and severity. While there was no significant difference in the genotypes of autistic and healthy subjects, there was a significant difference in the severity, particularly social communication, in the ARNT gene (SPN rs2228099), but not AhR rs2066853, polymorphism [130]. Although the underlying mechanisms were not investigated, alteration of the gonadal hormone balance mediated by regulating AhR was postulated and, thus, more genetic analyses are necessary. Additionally, a genetic variant of ARNT2 (SPN rs17225178) was associated with patients with Asperger syndrome, a subtype of autism that is not associated with delay in language or cognitive development [131]. Since ARNT is an AhR partner, it is highly suggested that exposure to environmental toxicants may affect the ASD.

Thai children and adolescents with ASD exhibited increased frequencies of clinically relevant polymorphisms of CYP1A1 at SNP rs1048943 and rs4646422 (30.3%), CYP1A2*1C rs2069514 (30.3%) and CYP1A2*1F (rs762551, 23.9%) [132]. These results suggest that polymorphism of AhR pathway genes plays an important role in the severity of autism, especially in regard to social communication. Sleep onset delay in children with autism is attributed to melatonin deficiency due to dysfunctional variation in genes related to the melatonin pathway [133]. One of the genes mediating melatonin metabolism is CYP1A2 and, to a lesser extent, CYP1A1, in which polymorphism of CYP1A2 has been linked to the susceptibility of ASD with combined disrupted sleep [134]. A significant decrease in CYP1A2 enzyme activities in children with autism was associated with significantly higher frequencies of three variant alleles CYP1A2*1C at SNP rs2069514, CYP1A2*4 at SNP rs72547516), and CYP1A2*6 at SNP rs28399424 [134]. Thus, elevated levels of melatonin results in loss of circadian rhythms and eventual loss of supplemental melatonin effectiveness. Further evidence supporting the role of CYP1A2 variations in ASD is the low urinary melatonin metabolite, 6-sulfatoxymelatonin, by CYP1A2 in mothers of children with autism compared to control mothers [135]. These studies collectively indicate that low CYP1A2-mediated melatonin deficiency is a risk factor and early indicator of ASD. 

**Table 2:** Impact of epigenetic and polymorphic changes by AhR/CYP1 on autism.

Mechanisms	Study Model	Study Design	Gene/Enzyme Changes	Effect on Autism	References
**DNA methylation**	Human	Autistic subject	↑ DNMT3a, b	↑ DNA damage	[100]
		Autistic men exposed prenatally to PCBs	↑ Methylation of CYP1B1/Cyp1A1	↑ Incidence of autism	[102]
			↓ Methylation of AhRR		[102]
		Newborns with maternal smoking during pregnancy	↑ Methylation of Cyp1A1	↑ Incidence of autism	[103,106]
			↓ Methylation of AhRR	hypomethylation in the early development period can persist for a long period	[103,104,106]
	Rats	Pre- & postnatal exposure to AhR activators	↓ DNA methylation ↓ DNMT1, 3a, 3b mRNA levels in brain	↓ Sp1↓ S-adenosyl methionine levels.	[107,108,109]
			↑ DNMT1 and ↑ ARNT	Delay in onset of puberty in males and affecting estrous cyclicity in females	[111]
	Mice	BTBR T+tf/J mice	↑ DNMT3a, b	↑ DNA damage and autism risk	[100]
**Histone modifications**	Human	HEK293 cells exposed to PCB Human post-mortem cerebellum autistic individuals	↑ Demethylation of H3K4me3 by Jarid1b ↑ DNMT3a and 3b	↑ Activation of androgen receptor transcriptional activity ↑ DNA oxidative damage genes 8-oxodG → ↑ autism incidence	[100,121]
	Rats	Valproic acid -induced autism in rats	↓ HDAC → hyperacetylation of histone	↑ Neurological birth defect	[115]
	Mice	BTBR T+tf/J mice	↑ DNMT3a and 3b ↔ H3K9ac, H3K56ac, H3K4me3, H3K9me3, H3K27me3, and H4K20me3 compared to the control	↑ DNA oxidative damage genes 8-oxodG → ↑ autism incidence	[100]
**MicroRNAs**	Mice	Prenatal exposure to TCDD	↑ miR-379	↓ Brain neuronal development → ↑ hypo-social behavior observed	[128]
			↓ let-7	Regulated neuronal stem cell proliferation	[128]
**Gene polymorphism**	Human	Autistic subjects	↑ ARNT gene (rs2228099), but not AhR rs2066853,	Significant difference in the severity, particularly social communication	[130]
			↑ ARNT2 (rs17225178)	↑ Association with Asperger syndrome	[131]
		Autistic children	*CYP1A1* rs1048943 and rs4646422 CYP1A2*1C (rs2069514) CYP1A2*1F (rs762551)	Associated in Thai children and adolescents with autism spectrum disorder	[132]
			↑ CYP1A2*1C (rs2069514), CYP1A2*4 (rs72547516), and CYP1A2*6 (rs28399424) → ↓ CYP1A2 activity → ↑ levels of melatonin	Loss of circadian rhythms and loss of supplemental melatonin effectiveness	[134]

## 5. The Involvement of AhR/CYP1 Pathways in Autism Development and Treatment by Drugs

### 5.1. Sulforaphane

Sulforaphane is a natural dietary compound, derived from many cruciferous plants, such as broccoli, cabbage, and cauliflower, with potent anticancer activity [136,137]. Previous placebo-controlled, double-blinded, randomized clinical trial, studies showed that sulforaphane treatment significantly reduced the symptoms of and improved the behavioral abnormalities in male ASD [136,138]. The cytoprotective effect of sulforaphane is mainly mediated by the activation of nuclear factor erythroid 2–related factor 2 (NRF2)-dependent antioxidant genes, such as NAD(P)H: quinone oxidoreductase-1 (NQO1), and heme oxygenase-1 (HO-1) [139,140]. In addition to its effect on the NRF2 pathway, it was shown that sulforaphane is a potent antagonist for AhR activation and CYP1A1 and CYP1A2 induction in human hepatoma HepG2 and breast cancer MCF-7 cells [141], and in rat precision-cut liver slices [142], suggesting that the AhR/CYP pathway could mediate sulforaphane’s protective effect on autism. Mechanistically, it could be postulated that sulforaphane inhibits AhR/CYP1 activation, causing DNA adduct and a DNA strand break [143]. This is supported by the observations that high levels of oxidative stress and oxidative DNA damage, such as 8-oxo-7-hydrodeoxyguanosine, 5-methylcytosine, and 5-hydroxymethylcytosinehave, have been reported in subjects with ASD [144], and in the cerebellum of a BTBR T+tf/J autistic mouse model [100]. A recent systematic review aimed to evaluate the therapeutic use of sulforaphane on patients with autism showed evidence that sulforaphane is an effective treatment option for treating ASD [145]. 

### 5.2. Valproic Acid

Valproic acid, or sodium valproate, is an archaic drug used to treat bipolar disorder and epilepsy with low safety margins [146]. Maternal exposure to valproic acid during pregnancy leads to development of autism-like behavior and in the offspring and childhood [147,148]. This effect seems to be only a property of valproic acid and no other antiepileptic agents as exposures to carbamazepine, oxcarbazepine, lamotrigine, and clonazepam monotherapy did not increase risks of childhood autism, which could be attributed to the difference in their structures [149]. Thus, valproic acid is a preferred model of studying autism in rodents [115,149,150]. An ASD gene-environment interaction study showed that approximately 130 ASGs are targeted by valproic acid [34]. However, some studies have linked valproic acid-induced autism and AhR as a postulated mechanism. Knowing that valproic acid is a HDA enzyme inhibitor that could alter histone structure and cause changes in the binding of transcription factors to DNA, valproic acid induces the expression of both AhR and CYP1A1 [151] through DNA methylation [152]. However, further studies are needed to investigate the role of the AhR pathway in valproate-induced autism.

### 5.3. Resveratrol

Resveratrol is a dietary compound with neuroprotective, anti-inflammatory and antioxidant properties. It is a known repressor of the AhR pathway [153]. Resveratrol and its methoxy derivatives are capable of downregulating AhR-related genes [154]. Interestingly, resveratrol, when administered prenatally, prevents social impairments in mice models induced with autism-like behavior using valproic acid [155]. It was also found to reverse cellular and behavioral sensory alteration in valproic acid-induced rat models of ASD [156]. Furthermore, treatment with resveratrol of rats exposed prenatally with progestin to induce autism caused dose-dependent reduction in core and autism symptoms, including neurological, sensory, behavioral, biochemical, and molecular deficits [157]. Importantly, resveratrol and its methoxy derivatives are capable of downregulating CYP1 genes [153,154]. Based on these results, it could be assumed that resveratrol is able to induce such positive changes in autistic behavior by suppressing the AhR pathway; however, more studies are needed to support this hypothesis.

### 5.4. Metformin

Metformin is a clinically used anti-hyperglycemic drug for treating diabetes mellitus type II. Wang et al., who studied the effect of administration of metformin treatments in BTBR mice, have demonstrated that metformin can enhance social interaction and decrease repetitive behaviors [158]. The study suggested that neonatal metformin treatment is capable of averting some of the classic behavioral symptoms commonly observed in patients with ASD. In addition, a recent study conducted on rats to examine the effect of metformin on valproic acid-induced autism-like behaviors has demonstrated that postnatal treatment with metformin improved the sociability index, spatial learning, and reference memory deficits through the attenuation of malondialdehyde and nitrite levels, AChE activity, and antioxidant enzymes activities in the rat hippocampus and prefrontal cortex [159]. In humans, the clinical studies on the beneficial effects of metformin are controversial. A recent study on seven individuals with fragile X syndrome (FXS), a subtype of ASD, treated with metformin showed significant improvement in irritability, social responsiveness, hyperactivity, language skills, and social avoidance, as compared to the control [160]. This protective effect of metformin could be attributed to downregulation of the mTOR/MEK/ERK pathway, which is overexpressed in FXS [160]. From the AhR/CYP1 perspective, the ability of metformin to inhibit AhR activation and CYP1A1 and CYP1B1 expression, leading to a decrease in the oxidative DNA damage [158,161], could also explain, to some extent, the protective effect of metformin in autism. However, more studies are needed to further explore the role of AhR. 

### 5.5. Haloperidol

Haloperidol is a first-generation antipsychotic drug, which has been in use for decades to treat attention deficit hyperactivity disorder (ADHD), aggression, withdrawal, un-cooperation, and stereotypy [162]. It has also been found beneficial for language training, irritability [163], and decreasing behavioral symptoms in children with autism [164]. However, its use is limited due to increased susceptibility to acute dystonic reactions and dyskinesia [165]. Haloperidol is one of the drugs that can act as a substrate to CYP1A2 [166,167]. There has been suggested involvement of a potent inhibitor of CYP1A2, fluvoxamine, in dramatically increasing serum concentrations of haloperidol [168]. However, not many studies have dived into the extent to which CYP1A2 is involved in the metabolism of haloperidol in patients with autism, suggesting the possible involvement of AhR in haloperidol effectiveness or toxicity. A dose-dependent increase in serum concentrations of haloperidol was observed with co-administration of fluvoxamine, a potent inhibitor of CYP1A2 [168,169]. These studies suggest the involvement of AhR in the metabolism of haloperidol, but more studies are encouraged to explore the involvement of AhR in the metabolism of haloperidol in patients with ASD. 

## 6. Conclusions and Remarks

Autism was earlier believed to be exclusively caused by genetic inheritance. Therefore, for more than two decades now, research on the etiology of ASD has been dominated by genetic studies. A wide range of genetic and environmental factors may contribute to the cause of this disorder. Mass industrialization and globalization have contributed to the increase in production and accumulation of toxicants and xenobiotics in the environment, leading to an increase in various diseases and developmental disorders. ASD incidence has been linked to an increase in pollution by numerous epidemiological studies and various reviews have comprehensive compilation of the effects of pollutants during the critical fetal and infant stages of development.

**Figure 3:**
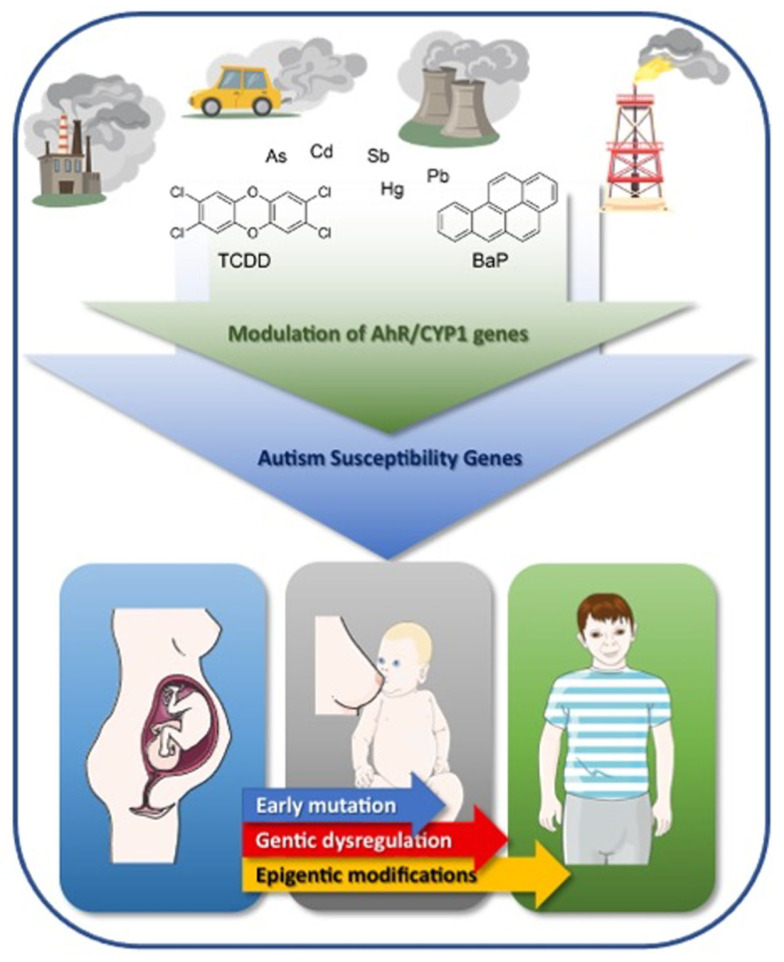
Graphical summary for the impact and mechanisms of environmental pollution in autism development.

It is well established that many xenobiotic compounds in the environment are capable of activating the AhR pathway by acting as ligands, binding to AhR protein, mediating the cascade of transcription and translation, resulting in proteins that help bioactivate the toxicants to their reactive entities. Although the AhR pathway has been well investigated in the past decade for its potential role in various diseases, including cancer, diabetes, and cardiotoxicity, there is a major void when it comes to ASD. This review explores the association of exposure to air pollutions and ASD and the molecular mechanisms (Figure 3). Evidence suggests the link between the AhR pathway and autism severity; however, few (to no) studies have been carried out to explore this possibility. Examining AhR and its role in autism may prove beneficial in understanding the etiology of the disease with deeper comprehension. For example, CYP1A1 and CYP1A2 have the ability to form DNA adducts, which may explain how sporadic de novo mutations arise in some individuals, leading to ASD development. AhR also alters DNA methylation, which has been suggested to cause ASD as well. Histone modification and gene polymorphisms are also suggested mechanisms by which environmental pollutants may increase the risk of ASD through the AhR pathway. Additionally, the current review sheds light on some novel, postulated AhR-mediated mechanisms for drugs that are currently used in autism.
